# Development and Characterization of Gentamicin-Loaded Arabinoxylan-Sodium Alginate Films as Antibacterial Wound Dressing

**DOI:** 10.3390/ijms23052899

**Published:** 2022-03-07

**Authors:** Abdulaziz I. Alzarea, Nabil K. Alruwaili, Muhammad Masood Ahmad, Muhammad Usman Munir, Adeel Masood Butt, Ziyad A. Alrowaili, Muhammad Syafiq Bin Shahari, Ziyad S. Almalki, Saad S. Alqahtani, Anton V. Dolzhenko, Naveed Ahmad

**Affiliations:** 1 Department of Clinical Pharmacy, College of Pharmacy, Jouf University, Sakaka 72388, Saudi Arabia; aizarea@ju.edu.sa; 2 Department of Pharmaceutics, College of Pharmacy, Jouf University, Sakaka 72388, Saudi Arabia; nkalruwaili@ju.edu.sa (N.K.A.); mmahmad@ju.edu.sa (M.M.A.); 3 Department of Pharmaceutical Chemistry, College of Pharmacy, Jouf University, Sakaka 72388, Saudi Arabia; mumunir@ju.edu.sa; 4 Institute of Pharmaceutical Sciences, University of Veterinary & Animal Sciences, Lahore 54000, Pakistan; adeel.masood@uvas.edu.pk; 5 Department of Physics, College of Sciences, Jouf University, Sakaka 72388, Saudi Arabia; zalrowaili@ju.edu.sa; 6 School of Pharmacy, Monash University Malaysia, Jalan Lagoon Selatan, Bandar Sunway 47500, Malaysia; muhammad.binshahari@monash.edu (M.S.B.S.); anton.dolzhenko@monash.edu (A.V.D.); 7 Department of Clinical Pharmacy, College of Pharmacy, Prince Sattam Bin Abdulaziz University, Riyadh 11942, Saudi Arabia; z.almalki@psau.edu.sa; 8 Department of Clinical Pharmacy, College of Pharmacy, Jazan University, Jazan 45142, Saudi Arabia; ssalqahtani@jazanu.edu.sa

**Keywords:** arabinoxylan, sodium alginate, wound healing, antibacterial dressing, drug delivery

## Abstract

Biopolymer-based antibacterial films are attractive materials for wound dressing application because they possess chemical, mechanical, exudate absorption, drug delivery, antibacterial, and biocompatible properties required to support wound healing. Herein, we fabricated and characterized films composed of arabinoxylan (AX) and sodium alginate (SA) loaded with gentamicin sulfate (GS) for application as a wound dressing. The FTIR, XRD, and thermal analyses show that AX, SA, and GS interacted through hydrogen bonding and were thermally stable. The AXSA film displays desirable wound dressing characteristics: transparency, uniform thickness, smooth surface morphology, tensile strength similar to human skin, mild water/exudate uptake capacity, water transmission rate suitable for wound dressing, and excellent cytocompatibility. In Franz diffusion release studies, >80% GS was released from AXSA films in two phases in 24 h following the Fickian diffusion mechanism. In disk diffusion assay, the AXSA films demonstrated excellent antibacterial effect against *E.coli, S. aureus*, and *P. aeruginosa.* Overall, the findings suggest that GS-loaded AXSA films hold potential for further development as antibacterial wound dressing material.

## 1. Introduction

A wound can be defined as a breakage in intact tissue (particularly skin), caused by physical, thermal, chemical, or mechanical trauma, or may result from complicated pathological conditions [1,2]. Immediately after injury, a programmed and complex wound healing process takes place, which can be categorized into four major interlinked healing stages: the hemostasis (clot formation), inflammatory (influx of inflammatory mediators and cells), proliferative (dermal cells proliferation, epithelialization, angiogenesis, and formation of granular tissues), and maturation (remolding and scar formation) stages [3,4,5,6]. The complete duration of healing may vary depending upon the nature of the wound [7,8]. Depending on the time required for complete healing, wounds can be classified into acute (1–12 weeks) and chronic (>12 weeks) wounds [8,9]. Wound infections, comorbidities (cancer, diabetes mellitus, obesity, etc.), malnutrition, and poor wound care are the main factors leading to chronic wounds that put a physical, psychological, social, and financial burden on patients [2,9,10]. Wound infections caused by bacteria (mainly *Escherichia coli*, *Staphylococcus aureus*, *Pseudomonas aeruginosa*, *Bacteroides fragilis*) are one of the most predominant reasons for the prolongation of wound healing (in the inflammatory stage) [1,11,12,13]. These bacteria form colonies at the wound site and may overcome the patient’s immunity, resulting in tissue damage and life-threatening consequences [14,15]. The development of an active dressing may prevent wound infections and augment the healing process.

Numerous kinds of dressing materials have been in human use since ancient times, with the primary aim being to cover the wound to provide protection against the environment and prevent bleeding and further injury [5,12,16]. Wound dressing in current practice can be broadly classified into dry/traditional (cotton, gauze, bandage) and moist/modern (films, foams, hydrogels, sponges, hydro actives, hydrofibers) dressings [8,17]. Dry dressings are still in clinical practice despite possessing the main limitations of wound dehydration and risk of infection [12,18,19]. Conversely, modern dressings augment wound healing by preventing dehydration and facilitating cell proliferation [12,17]. Additionally, antimicrobial agents can be incorporated into the polymeric dressings for delivery into the wound bed to prevent wound infections [8,20]. In addition to providing a moist environment and promoting healing, an ideal dressing should be biocompatible, mechanically durable, and flexible, allow water and gaseous exchange, and possess antimicrobial properties [5,8,16,21]. Among the modern dressings, polymeric films offer the advantage of gas permeability, moisture transmission, wound inspection (due to transparency), impermeability to microbes, ease of application to joints (due to flexibility), and drug incorporation and delivery to wound site [8,22]. However, difficulty in removing them from the wound, limited inherent antimicrobial effect, and lesser absorption of the exudate are the major limitations of the films [8]. Therefore, researchers are keen to develop films with novel combinations of polymers and antibacterial agents to provide optimal healing conditions [23,24].

Several natural, semi-synthetic, and synthetic polymers have been explored to develop film dressings, as reviewed recently by Savencu et al. [8]. Among the natural polymers, alginate (sodium and calcium salts), a linear unbranched polysaccharide (containing mannuronic and guluronic acid) found in brown algae, is commonly employed in wound dressing formulations owing to its excellent physicochemical, biocompatible, hemostatic, water sorption, and film-forming properties [8,17,25,26,27,28,29,30]. Moreover, SA may also trigger macrophages and the release of inflammatory mediators that augment the healing process [29]. Numerous alginate-containing dressing materials are commercially available; recent research on antibiotics-loaded alginate dressings has demonstrated their potential to prevent infections [26]. Most of these dressings were prepared by a combination of sodium alginate (SA) and other natural or synthetic polymers to improve various features required in the dressings [25,27,28,29,30]. 

Arabinoxylan (AX), isolated from the husk of *Plantago ovata* (psyllium) seed husk (PSH), is a branched polysaccharide that mainly contains xylose backbone and arabinose side chains with a minor percentage of rhamnose and uronic acids [31]. AX is an attractive material for biomedical applications owing to its biocompatible, film-forming, water absorption, and drug delivery characteristics [32,33]. In our previous studies, AX and AX-gelatin (AX-GL) films exhibited potential for film dressing application [32,33]. However, there is a need to improve the features of these AX-based films, especially their mechanical stability in the wound exudate. Previously, it was found that AX and AX-GL films rapidly uptake water, expand in the simulated wound environment, and start deteriorating. Preparing composite films is a simple and efficient approach to improving polymeric films’ physicochemical characteristics [29]. Therefore, in this work, AX was combined with SA to improve the physicochemical and wound healing properties of AX-based films. The combination of AX with SA is expected to improve the mechanical stability of the AX-based films in a moist wound environment owing to the hydrogen bonding interaction between AX and SA. Moreover, adding the SA in AX-based dressings formulation will also augment the healing process by triggering the release of inflammatory mediators (as discussed above). However, both AX and SA lack antibacterial activity; therefore, gentamicin sulfate (GS), a broad-spectrum aminoglycoside, is incorporated into AXSA film dressings due to its efficacy again bacteria causing wound infection [34,35]. To the best of our knowledge, GS-loaded films composed of AX and SA have not been previously reported. 

In this study, novel GS-loaded AXSA films were fabricated for application as an antibacterial wound dressing. The thickness, mechanical properties, morphology, water transmission, water uptake, chemical nature, thermal degradation behavior, drug release, antimicrobial, and cell viability were investigated to assess the suitability of AXSA films for antibacterial wound dressing application.

## 2. Materials and Methods

### 2.1. Materials

Sodium alginate (SA) (from brown algae) (Mol. Wt. 120,000–190,000, viscosity: 5 to 25 cps, M/G ratio: 1.56), glycerol (Gly). PBS tablets, CaCl_2_ granules (anhydrous), pen-strep solution (containing 10,000 penicillin units and 10 mg of streptomycin per mL), cellulose acetate membranes (0.45 μm, 25 mm), MTT (3-(4,5-dimethylthiazol-2-yl)-2,5-diphenyl-tetrazolium bromide), polystyrene petri dishes (10 cm × 20 mm), and gentamicin sulfate were procured from MilliporeSigma (St. Louis, MO, USA). MRC-5 cells (ATCC CCL-171) were cultured in RPMI-1640 culture medium supplemented with FBS (Nacalai Tesque, Kyoto, Japan). Three bacterial strains *S. aureus* (ATCC 25923), *E. coli* (ATCC 8739), and *P. aeruginosa* (ATCC 9027) were all cultured in Mueller-Hinton broth (MHB) and Agar (Sigma-Aldrich, MO, USA). Arabinoxylan (AX), containing Xyl*p* (72.5%), Ara*f* (21.8%), Rha*p* (2.3%), and uronic acids (1.4%) was isolated from *Plantago ovata* (PO) seeds husk (Mol. Wt. 364,470) as reported previously [31,32].

### 2.2. Fabrication of AXSA Films

The AXSA films were fabricated by solvent casting technique [33]. Briefly, uniform dispersions of SA (3.5, 3, 2.5, and 2% *w*/*w*) were prepared in distilled water by high-speed stirring (1000 rpm) at 40 °C for 12 h. Then, AX (1.5, 2, 2.5, and 3% *w*/*w*) and glycerol (2.5%) were added to SA dispersion and stirred until homogenized gel was obtained. The resultant AXSA film casting gels were sonicated to eliminate air. After that, to cast blank AXSA films, 25 g of AXSA gels were poured into polystyrene plates and kept for drying in an oven at 40 °C for 48 h. While for GS-loaded AXSA films, GS (0.1% *w*/*w*) was added in homogenized AXSA gels and films were cast as described for blank films. After drying, films were peeled from plates, and their physical appearances (transparency, flexibility/foldability, smoothness) were visually observed. The films were kept in a desiccator until further analysis. The composition and codes of AXSA film formulations are displayed in Table 1.

### 2.3. Characterization of AXSA Films

#### 2.3.1. Film Thickness

The cross-sectional thicknesses of all dried films were measured at five randomly selected points. The thickness measurements were performed using a micrometer with 0.01 mm accuracy (APT measuring instruments, Omaha, NE, USA).

#### 2.3.2. Water Vapor Transmission Rate (WVTR)

The WVTR of AXSA films was measured following the previously reported procedure with some modifications [32]. Briefly, AXSA films were cut into ~2.5 cm diameter and placed on openings of glass vials containing 2 g of CaCl_2_ (desiccant). Then, caps with a 1 cm opening were fitted on the film containing vials. Then, these vials were weighed and kept in a desiccator at 85 ± 3% RH and 25 ± 2 °C. The change in the weights of the vials was noted at constant time intervals up to 24 h. WVTR was calculated according to the formula described earlier [32]. 

#### 2.3.3. Mechanical Properties

Mechanical tests were performed using a 5 kN load cell (LS5) tensile testing machine (Lloyd instruments, West Sussex, UK). For this purpose, dumbbell-shaped specimens (0.5 cm width and 3 cm length) of AXSA films were prepared. To protect the film from damage due to grip, the film’s area in contact with the grip was covered with double adhesive tape. The films were stretched at a crosshead speed of 10 mm·min^−1^ until breakage. Finally, tensile strengths (TS) and percent elongations at break (% EAB) were calculated according to the formula described previously [36].

#### 2.3.4. Surface Morphology

The surface morphology of gold sputter-coated AXSA films (with and without GS) was observed using a Quanta 250-FEG scanning electron microscope (SEM) (FEI, OR, USA) at 5 kV operating voltage and 1000× and 2000× magnifications. 

#### 2.3.5. FTIR Spectroscopy

ATR-FTIR spectroscopic analyses were performed with an FTIR-7600 spectrophotometer (Lambda-Scientific, Marion, South Australia, Australia). Transmittance spectra were recorded at 4 cm^−1^ resolution in the wavenumber range of 4000 to 550 cm^−1^.

#### 2.3.6. X-ray Diffraction

X-ray diffraction (XRD) analyses were performed with Shimadzu X-ray diffractometer (MAXima_X XRD-7000, Kyoto, Japan) using CuKα radiations. The X-ray diffractograms were recorded in the 2*θ* range of 10 to 80° by operating at 30 mA, 40 kV, and 2° min^−1^ scanning speed.

#### 2.3.7. Thermogravimetric Analyses (TGA)

The TGA analyses were performed with Shimadzu TGA-50 analyzer (Kyoto, Japan). TG curves were obtained by heating weighed AXSA films from 30 to 600 °C at 20 °C. min^−1^ heating rate and 20 mL. min^−1^ nitrogen gas flow.

#### 2.3.8. Differential Scanning Calorimetry (DSC)

The DSC curves were acquired on DSC3 STAR instrument (Mettler Toledo, Columbus, OH, USA), operated at 20 °C. min^−1^ heating rate. The changes in the heat flow of AXSA films were recorded from 30 to 450 °C under 20 mL.min^−1^ nitrogen gas flow.

#### 2.3.9. Expansion Profile

The exudate absorbing profile of the AXSA films was investigated by measuring the expansion of films over a gelatin solution (4 g. 100 mL^−1^). Briefly, to create an environment similar to exudating wounds, 25 mL of gelatin solution was allowed to solidify in the polystyrene plates for 18 h at 25 °C. Then, pieces of AXSA films (~25 mm diameter) were placed over solidified gelatin, and expansion in the size of the films was measured using a ruler at predefined intervals of time. The percent expansion was calculated according to the previously described formula [29].

### 2.4. Gentamicin Release Profile and Kinetics

The release profile of GS from GF2 and GF3 films was studied with DHC-6T Franz diffusion cell (Logan-Instruments, Somerset, NJ, USA) using 10 mL release media (PBS, pH 7.4) in the receiver compartment. Cellulose acetate membranes were placed over the receiver compartment, and then weighed GS-loaded AXSA films (~1.5 cm diameter) were mounted over these membranes. GS release experiments were performed at 37 °C, with constant stirring of the release media. Aliquots were withdrawn from the receiver compartment at pre-programmed time points. The concentration of GS in the aliquots was determined according to the *o*-phtaldialdehyde derivatization method using a Genesys 10s Uv-Vis spectrophotometer (Thermo Fisher Scientific, Waltham, MA, USA). The GS release profile was plotted as percent cumulative release versus time [37]. 

To determine GS release kinetics and mechanism of release from AXSA films, the following kinetic models were fitted to the release data:
Zero order$Q_{t}=Q_{0}+K_{0}t$(1)First order$InQ_{t}=InQ_{0}+K_{1}t$(2)Higuchi$Q_{t}=K_{H}t{\;}^{1/2}$(3)Korsmeyer–Pappas$Q_{t}=K_{k}\;t{\;}^{n}\;$(4)
where *Q_t_* and *Q*_0_ are the amounts of GS released from AXSA films at time *t* and 0, while *K*_0_, *K*_1_, *K_H_*, and *K_k_* represent kinetic constants and *n* is the release exponent.

### 2.5. Antibacterial Effect of AXSA Films

Antibacterial activities of the GS-loaded (GF2 and GF3) films were estimated using the agar disk (filter paper) diffusion method. For this purpose, AXSA films were cut into ~6.8 mm with filter paper backing and sterilized by UV exposure for 60 min. Autoclaved MHA (40 mL) was poured into glass petri plates and allowed to solidify. Then, standardized inoculums (20 µL) of bacterial strains (*S. aureus*, *E.coli*, *and P. aeruginosa*) were uniformly spread over MHA using a glass spreader. After that, GS-loaded AXSA films (GF2 and GF3), GS standard, and blank AXSA film (F2) were placed over the bacterial cultures, and plates were incubated for 24 h at 37 °C. Clear zones of inhibition formed around the samples were measured.

### 2.6. Indirect Cell Viability Assay

The effect of AXSA films on the viability of MRC-5 cells was investigated by indirect MTT assay. For this purpose, extracts of the blank (F2) and GS-loaded (GF2) AXSA films were prepared by incubating weighed films in culture media for 24 h under aseptic conditions, as reported previously [38]. MRC-5 cells were cultured in RPMI-1640 with 10% *v*/*v* FBS, 1% *v*/*v* pen-strep at 37 °C in humidified 5% CO_2_ incubator. Then, cells were harvested and seeded in 96-well plates (~7500 cells per well) and incubated for another 48 h. After that, culture media in the 96-well plates was replaced with AXSA films extract, except the control (untreated) well, and incubated for 48 h. Finally, according to the previously reported method, an MTT assay was performed to determine the percent cell viability (% CV) [39]. 

### 2.7. Statistical Analysis

Statistical analysis of the data was performed using GraphPad Prism software. The differences were considered statistically significant when *p* was found to be less than 0.05 by one-way ANOVA. The results are expressed as mean ± standard deviation (SD).

## 3. Results and Discussion

### 3.1. Fabrication of AXSA Films

The AXSA gels containing 5% (*w*/*w*) AX and SA, prepared by high-speed stirring, were easy to pour, and free from air bubbles and polymer aggregates. This 5% (*w*/*w*) gel concentration for casting AXSA films was selected based on previous studies on alginate composite films [29]. However, when AX concentration increased above 3% in AXSA gels, the gel became highly viscous and difficult to pour [33]. Therefore, F1, F2, F3, and F4 gels were used to cast blank AXSA films. The resultant AXSA blank films were visually observed for their appearance and suitability for wound dressing application. The pictures of the blank and GS-loaded AXSA films are shown in Figure S1. Overall, all the AXSA films were transparent, smooth, flexible (foldable), and free from air bubbles. There was no significant difference in GS-loaded films’ physical appearance compared to blank AXSA films of the same concentration. However, F1 films were thin and difficult to peel from the plates. Therefore, F2, F3, and F4 films were selected for further studies. The transparency and foldability of the dressing are important parameters as transparency allows observation of the healing wound while foldability facilitates dressing of wounds in bending on parts of the body [29]. To study the effect of the plasticizer on the handling of the films, AX (3% *w*/*v*), SA (3% *w*/*v*), and AXSA (AX 2% and SA 3%) films without glycerol were prepared and compared with F2 film (with 2.5% glycerol), as shown in Figure S2. These results demonstrated that the dried films (without glycerol) were transparent but not flexible (fractured upon folding). Therefore, the films without plasticizers were not considered for further characterization for wound dressing application.

Gentamicin sulfate (0.1%) was incorporated into AXSA film formulation to fabricate GS-loaded films. The selection of GS dose was based on the previous reports where 0.1% GS was reported to be safe and effective for wound healing applications [14,40,41]. Moreover, topical formulations (creams and ointments) containing 0.1% GS are commercially available for topical application and considered non-toxic [40]. The total GS-loaded in 10 cm (diameter) AXSA films is 25 mg.

### 3.2. Characterization of AXSA Films

#### 3.2.1. Thickness

**Figure 1:**
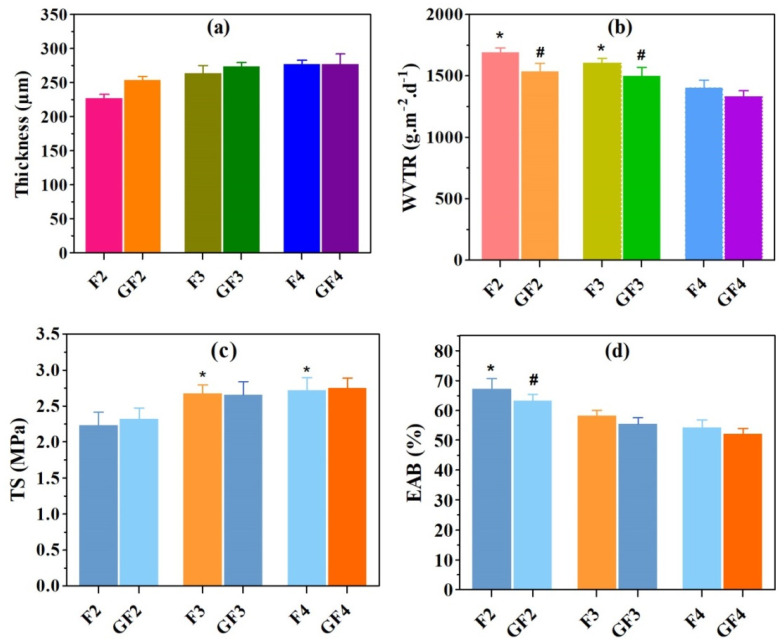
Results of (**a**) thickness, (**b**) water vapor transmission rate (WVTR), (**c**) tensile strength (TS), and (**d**) elongation at break (EAB) of the arabinoxylan (AX)-sodium alginate (SA) films (mean ± SD, *n* = 3). * and ^#^ show statistically significant differences (*p* < 0.05) from parallel blank and gentamicin (GS)-loaded films.

The mean thicknesses of F2, GF2, F3, GF3, F4, and GF4 AXSA films were 227 ± 6, 253 ± 6, 263 ± 11, 273 ± 6, 276 ± 14, and 276 ± 9 µm, respectively, as shown in Figure 1a. These results indicate that there was no significant difference in the thickness of the AXSA films. However, a slight increase in film thickness was found with increasing AX concentration and the addition of the GS in the film formulations. According to previous reports, the major factors that control the thickness of polymeric films include composition, the weight of the gel used for film casting, the interactions between the polymers, drying conditions, and the flatness of the drying surface [25,29]. Since the total amount of AX and SA (5%) was constant in AXSA films, the slight increase in the thickness of the AXSA film formulations was due to AX, which has previously been reported to form thick films [33]. Despite containing higher total polymers contents (AX + SA), the thickness of the AXSA films was lower than previously reported AX and AX-GL films, which may be due to lower casting volume and hydrogen bonding between the AX and SA. Nevertheless, the thickness of the AXSA films was in the range of the average thickness of the human skin (200 to 500 µm). Therefore, it can be suggested that these AXSA films will be suitable for application to skin wounds, as thin films are more comfortable for patients [25].

#### 3.2.2. Water Vapor Transmission Rate (WVTR)

The results of the WVTR of the AXSA films (blank and GS-loaded) are presented in Figure 1b. The average WVTR values for the F2, GF2, F3, GF3, F4, and GF4 films were 1687 ± 41, 1537 ± 69, 1604 ± 39, 1498 ± 65, 1403 ± 59, and 1326 ± 57 g·m^−2^.day^−1^, respectively. In blank AXSA films, WVTR of the F2 and F3 films was significantly higher than in F3 films, while in GS-loaded films, WVTR of the GF2 and GF3 films was significantly higher than that of GF4 films. Moreover, the WVTR of GS-loaded films was less than that of the blank AXSA films, but the difference was statistically insignificant. The WVTR of the film dressings usually depends on the chemistry of the polymers, thickness, composition, and porosity [29]. The WVTR results of the AXSA films indicate that this parameter decreases in tandem with decreasing SA concertation in films. Since the thickness of the AXSA films was not significantly different, the decrease in WVTR with smaller quantities of SA in film can be explained by the decrease in the number of carboxylic groups interacting with water molecules [42]. Moreover, an increase in AX in the film formulations might result in a denser hydrogen bonding network between the -OH groups of AX and the -COOH groups of SA, thus forming films with a lower WVTR [43]. Similar trends in the decreasing WVTR of the SA-containing blended films have been reported previously [42,43]. The WVTR of the AXSA films was higher than previously reported values for AX-GL films; this can be attributed to the lower thickness of the AXSA films [32]. The WVTR is vital in developing a wound dressing aimed for drug delivery. The WVTR of dressing for clinical application ranges between 139 to 10,973 g·m^−2^·day^−1^, and the WVTR of injured skin may be up to 4274 g·m^−2^·day^−1^ [44]. Hence, the WVTR of our AXSA films reported here is adequate for wound dressing application. 

#### 3.2.3. Mechanical Properties

The mechanical properties (TS and EAB) of the AXSA films are presented in Figure 1c,d. The TS of the AXSA films ranged between 2.31 MPa and 2.75 MPa, while the EAB varied from 54% to 67%. Overall, the TS of the AXSA films increased, and EAB decreased with increasing AX concentration in the films. Moreover, the TS of the GS-loaded AXSA films were slightly more than that of blank AXSA films. The TS of F3 and F4 films were significantly higher than the TS of F2 films, while the EAB of the F2 was significantly higher than that of F3 and F4 films. These differences in the mechanical properties suggest that intermolecular interactions between AX and SA resulted in the increased rigidness of the films and decreased flexibility [29]. The mechanical properties of the film dressings were also reported to be influenced by the concentration of plasticizer (glycerol) and the total amount of solids in the films [29]. However, the amount of plasticizer and the total amount of polysaccharides (AX + SA) was the same in all films; therefore, their influence on the mechanical properties of the AXSA films was not prominent. The F2 and GF2 films (with 2% AX) were not damaged during mechanical testing as compared to the previously repowered AX films, where films prepared by 2% AX were damaged during mechanical testing, suggesting that the addition of SA improved the mechanical stability of the films [33]. The TS and EAB of the AXSA films were lesser than earlier reported AX-GL films that may be due to lesser thickness and lower amount of plasticizer in of AXSA films [32]. The TS and the flexibility of the wound dressing are essential because an ideal material for wound dressing should be strong enough to endure the pressure and stretch during application and flexible for sustaining possible stretch during body movements [21,36]. According to the literature, the TS of the human skin varies from 2.5 to 16 MPa [21]. Hence, the TS of the prepared AXSA films is adequate for wound dressing applications. 

#### 3.2.4. Surface Morphology

**Figure 2:**
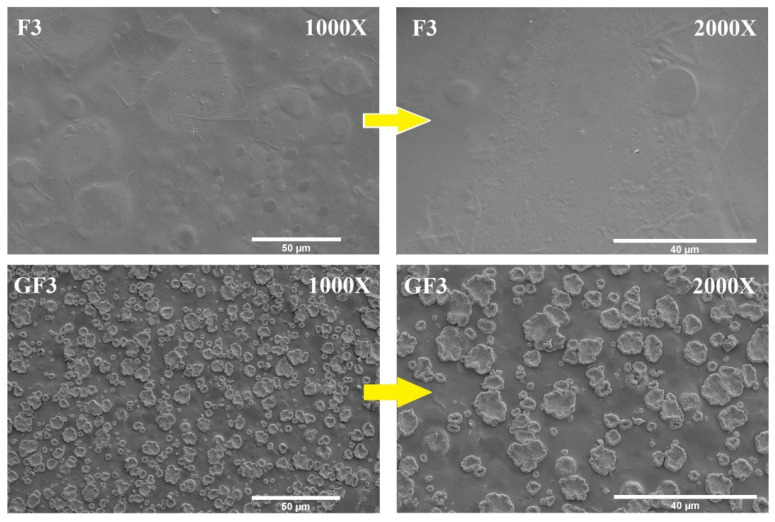
Surface morphology of blank (F3) and GS-loaded (GF3) films.

SEM was used to investigate the surface microstructure and smoothness of the blank and GS-loaded AXSA films, as presented in Figure 2. The surface morphology of the blank films (F3) was compact and smooth, without any cracks, aggregation of the AX or SA, or particles (Figure 2). This compactness and smoothness of the surface structure indicates the physical compatibility and high miscibility between the AX and SA [43]. Conversely, small particles were observed in the GS-loaded (GF3) film (Figure 2), which were uniformly distributed throughout the surface of the films. Since the major difference between the composition of the GF3 and F3 films is the presence of GS in GF3 film. Therefore, particles observed in SEM images of GF3 film might be GS particles located at the film’s surface. Similar particles were previously found on the surface of AX and AX-GL films after loading the GS in films [32,33]. The uniform distribution of drugs in films formulations is essential from the drug delivery perspective as it ensures the uniformity of the drug dosage [32,33].

#### 3.2.5. FTIR Spectroscopy

**Figure 3:**
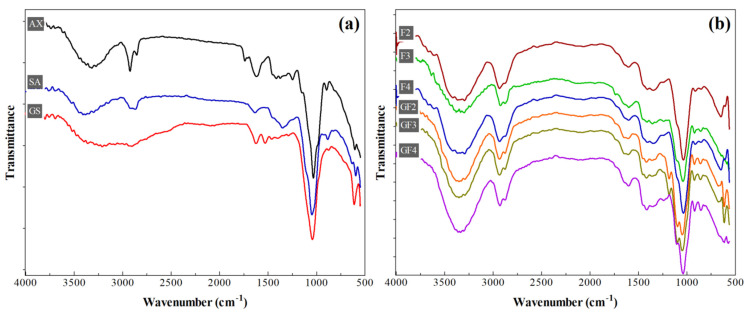
ATR-FTIR spectra of (**a**) pure components and (**b**) AXSA films.

The ATR-FTIR spectra of AX, SA, GS, and AXSA films are presented in Figure 3. Characteristic bands of the polysaccharide functional groups were present in the spectra of AX and SA (Figure 3a). In spectra of both AX and SA, peaks attributed to the –OH, –CH_2_, –COOH, C–O–C (glycosidic linkage), and C–O–H (primary alcohol) are present at around 3650–3300 cm^−1^, 2930 cm^−1^, 1660 cm^−1^, 1450 cm^−1^, 1160 cm^−1^, and 1055 cm^−1^, respectively [30,39]. The spectrum of GS exhibited major transmittance bands of –OH and Amide A (3650–2550 cm^−1^), Amide I (1645 cm^−1^), Amide II (1540 cm^−1^), and C–O–C (1050 cm^−1^) [31]. On the other hand, all characteristic peaks of both polysaccharides (AX and SA) were present in the spectra of both blank and GS-loaded AXSA films (Figure 3b). However, the peaks attributed to carboxylic groups were shifted from 1660 cm^−1^ and 1450 cm^−1^ to 1620 and 1425 cm^−1^, indicating the hydrogen bonding interaction between polysaccharides. The shift in the vibrational frequency of hydroxyl groups from 3423 cm^−1^ (SA) and 3360 cm^−1^ (AX) to 3350 cm^−1^ (in films) confirms that hydrogen bonding is present in the films due to the interaction between hydroxyl, carboxylic, and amide groups of AX, SA and GS [39]. The hydrogen-bonding interactions contribute to increasing the tensile strength of the AXSA films (Section 3.2.3). Moreover, in the spectra of AXSA films, some functional group peaks were broad due to the overlapping of the polysaccharide peaks with glycerol and GS peaks (in GS-loaded films). These findings of the FTIR spectra of AXSA films suggest that the film components (AX, SA, GS, and glycerol) were finely blended, chemically compatible, and no chemical reaction occurred during the fabrication of the AXSA films.

#### 3.2.6. X-ray Diffraction

**Figure 4:**
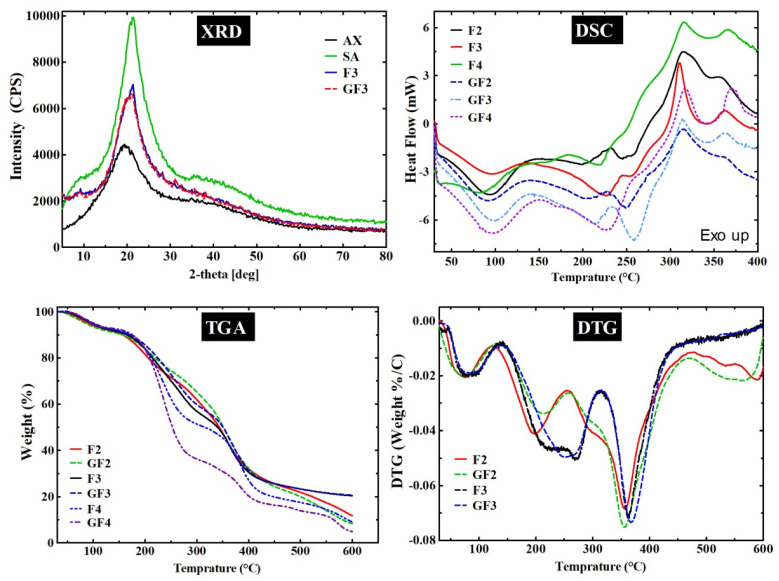
XRD, DSC, TGA and DTG analyses of the AXSA films.

The results of the XRD analyses of AX, SA, F3 (blank film), and GF3 (GS-loaded film) are presented in Figure 4. SA is semi-crystalline and exhibits characteristic peaks at around 2*θ* 13°, 56°, 20.6°, 20.1°, 29° and 36.4° [45]. The purpose of performing XRD analyses was to investigate the effect of combining film components on the semi-crystalline nature of the SA in AXSA films. The XRD pattern of SA had peaks between 2*θ* 13° to 38°, whereas AX did not show any sharp peaks attributed to its amorphous nature. The intensities of the semi-crystalline peaks of the SA were reduced in the AXSA films (F3 and F4). This reduction in the peak intensities may originate from the hydrogen bonding interaction of the SA with other amorphous components of the films (AX, Gly, and GS). Previously, Ramakrishnan et al. 2021 suggested a similar decrease in the SA crystallinity due to hydrogen bonding for SA/gum kondagogu blended films [43].

#### 3.2.7. Thermogravimetric Analyses (TGA)

The thermal degradation and stability of the AXSA films were investigated by TGA. The TG and DTG curves of the blank (F2, F3 and F3) and GS-loaded (GF2, GF3 and GF4) films are presented in Figure 3, while Figure S2 shows the results of the TGA of film components (AX, SA, Gly, and GS). In the first step of thermal degradation, all tested films and pure components (except Gly) exhibited weight loss up to ~120 °C, which was attributable to the loss of free water molecules and water interacting with hydroxyl and carboxylic groups [27]. Glycerol underwent characteristic single-step degradation at temperature range (*T_range_*) ~125–300 °C (weight loss (∆W) = 99.3%) with maximum weight loss temperature (*T_max_*) at 256.2 °C. The TG and DTG curves show that AX exhibited degradation at *T_range_* < 120 °C (∆W = 5.1%), 260–320 °C (∆W = 15.8%, *T_max_* = 291), 325–395 °C (∆W = 28.9%, *T_max_* = 354 °C), and > 395 °C (∆W = 21.7%) attributed to the loss of water, AX backbone and complete pyrolysis [32]. On the other hand, SA showed thermal decomposition at *T_range_* < 105 °C (∆W = 5.7%), 265–415 °C (∆W = 71.3%, *T_max_* = 330 °C), and >415 °C (∆W = 12.5%) due to water loss, fragmentation of polysaccharide backbone of SA and complete degradation [27]. Similarly, GS underwent thermal degradation at *T_range_* < 120 °C (∆W = 13.7%), 245–295 °C (∆W = 13.4%, *T_max_* = 274 °C), 295–370 °C (∆W = 25.9%, *T_max_* = 330 °C), and >370 °C (∆W = 35.5%), which shows that thermal decomposition of GS starts at 245 °C [46]. 

The F2 and GF2 films exhibited similar thermal degradation events at Trange <120 °C (∆W up to 8%), 125–245 °C (∆W up to 19.8%), 250–430 °C (∆W up to 47.1%), and >430 °C (∆W up to 18.8%). Similarly, F3 and GF3 films show similar degradation events at Trange <120 °C (∆W up to 7.2%), 140–310 °C (∆W up to 36.6%), 310–410 °C (∆W up to 28.8%), and >410 °C (∆W up to 20.6%). These results suggest that AXSA films undergo four main events of thermal degradation corresponding to the degradation events of the individual components of these films. However, the effect of the composition of the AXSA films was observed in the TGA of the films. In F3 and GF3 films, the second degradation event was broader than F2 and GF2 due to the higher AX contents in the F3 and GF3 films, resulting in the merging of the degradation step for Gly and the first step for AX. Similarly, the second degradation step was slightly broader in GS-loaded films than in the blank films due to the merging of degradations steps for Gly and the first degradation step of GS in GS-loaded films. The TG curves of F4 and GF4 also exhibited similar four-step thermal degradation behavior. It was observed that due to the plasticizer’s effect, the onset temperature for polysaccharide (AX and SA) degradation shifted to a lower temperature in films vs. pure polysaccharides. These shifts in the TG and DTG curves for the AXSA films indicate the miscibility of the components [47]. These findings for the thermogravimetric analysis of the AXSA films are consistent with previous studies on AX and SA-based films [27,32,33].

#### 3.2.8. Differential Scanning Calorimetry (DSC)

The results of the DSC analyses of the AXSA films (F2, F3, F4 GF2, GF3, and GF4) are depicted in Figure 4, while DSC curves of AX, SA, and GS are given in Figure S4. The DSC curves of the tested samples exhibited endotherms from 60 °C up to ~125 °C, indicating the removal of the water molecules. SA exhibited prominent exotherm at 297 °C, corresponding to the thermal degradation of the SA polysaccharide backbone as discussed above [27]. The DSC thermogram of AX showed characteristic exotherms at 290 and 312 °C (due to polysaccharide degradation), while a melting endotherm was found in the DSC of the GS at ~250 °C, which are in close agreement with the previous report [32]. On the other hand, two prominent endotherms were found in the DSC curves of the tested AXSA films at ~95 °C (water loss) and ~210 °C (due to glycerol). However, in DSC GS-loaded GF2 and GF3 films, a third endotherm was present at ~245 and 250 °C, respectively, attributed to the melting of GS. The second endotherm was broader in the DSC curve of the GF4, which might result from merging the endotherms due to glycerol and melting of GS. The exothermic peaks due to degradation of SA and AX were merged in the AXSA films and are present at ~310 °C. The DSC curves of the AXSA films did not show any significant shift in the DSC peaks of the components of the film. These findings of DSC analysis are consistent with previous studies of AX- and SA-based films and suggest that components of the AXSA films were highly miscible, and their stability was not affected during the casting process [27,32].

#### 3.2.9. Expansion Profile

**Figure 5:**
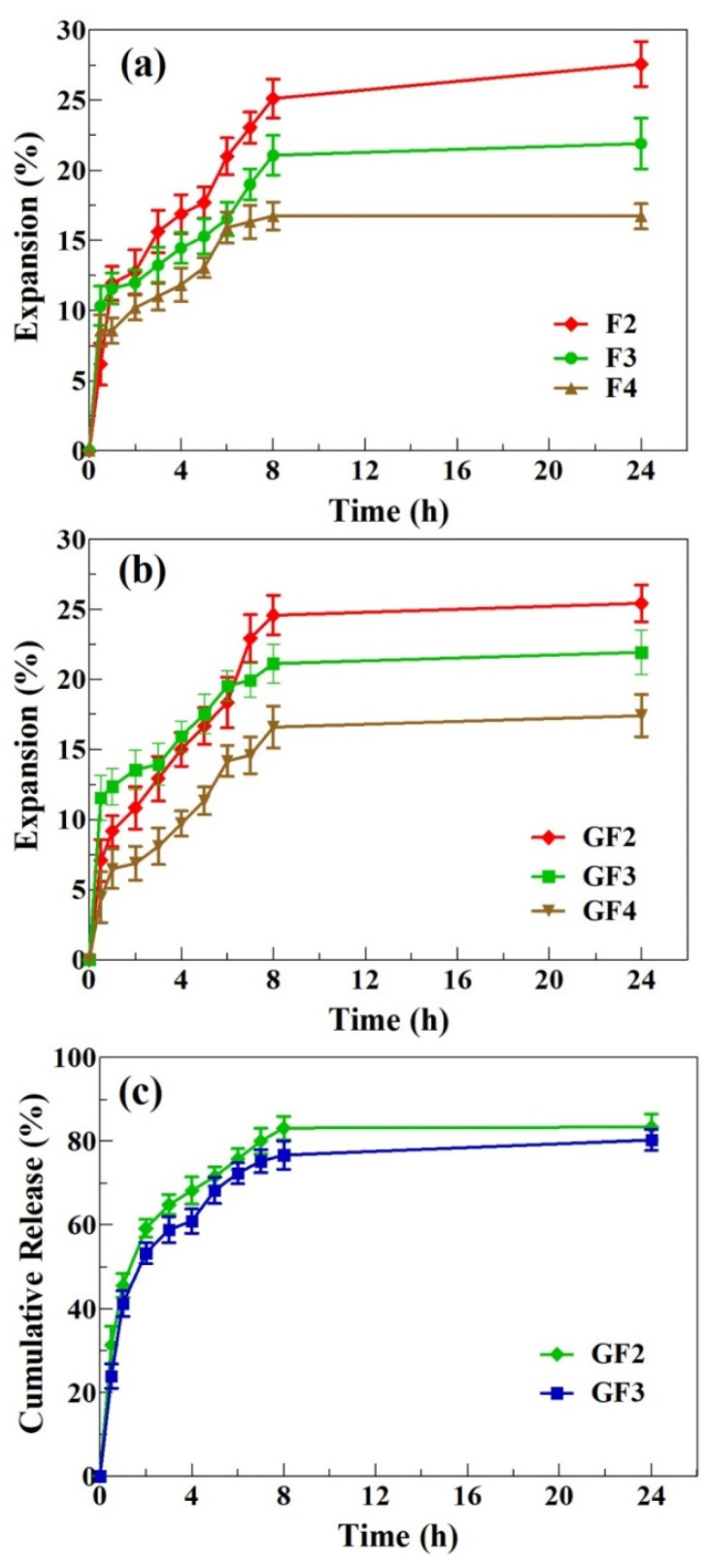
Expansion profiles of blank (**a**) and GS-loaded (**b**) AXSA films; and (**c**) drug release profile (mean ± SD, *n* = 3).

Water uptake (or hydration) is a vital characteristic of polymeric materials designed for wound dressing application. After application to the wounds, the dressings should absorb water (exudate) and expand in size while maintaining their integrity and shape [48]. Hydration also plays a key role in the dissolution of the drugs entrapped in the films [38]. Therefore, the expansion profiles of the AXSA films were determined using the gelatin model [32], and the results are shown in Figure 5a,b.

The expansion profile of AXSA films shows that films hydrated (uptake water) and expanded rapidly during the first hour of contact with solidified gelatin. After that, the expansion of the AXSA films slowed down. Among the blank AXSA films, F2 exhibited the highest expansion, followed by F3 and F4, respectively. Similarly, the expansion of GF2 films was highest among the GS-loaded AXSA films. Moreover, all AXSA films maintained their shape after 24 h. Higher expansion of the F2 films can be attributed to greater SA content in these films. The carboxylic group of SA converts to carboxylate ions in water, resulting in electrostatic repulsion among the polymeric chains, leading to the film expansion [49]. Therefore, higher expansion was observed in films containing more SA. As discussed above, an increase in the AX concentration in the films results in hydrogen bonding between the polymers, leading to the formation of a denser network and a decrease in free carboxylate ions available for interaction with water [50]. These factors lead to a further reduction in the expansion of the F3 and F4 films. The expansion behavior of AXSA films was in close agreement with previously reported simvastatin-loaded SA-pectin films [29]. However, the expansion percentage of the AXSA films was less than that previously reported for pure AX and AX-GL films, which can be explained by the hydrogen bonding interaction in the AXSA films [32,33]. Moreover, the AXSA films were intact (maintained their shape) throughout the expansion study compared to previously reported AX and AX-GL films, which started degrading after 5 to 6 in the expansion study [32,33]. Nevertheless, the expansion profile of AXSA films suggests that they hold the capacity to absorb the wound exudate while maintaining their shape and are suitable for wound dressing application [30].

### 3.3. Gentamicin Release Profile and Kinetics

The GS release profile from AXSA (GF2 and GF3) films, investigated by Franz diffusion cell, is presented in Figure 5c. The GF2 and GF3 films were selected for the antibiotic release experiment owing to their better characteristics (EAB%, WVTR and expansion profile) for wound dressing application. The release study results show that GF2 and GF3 released 45% and 41% of the incorporated GS during the first hour of the release study. The GS release became slower after that, and reached equilibrium by 24 h. The maximum cumulative release after 24 h was 83.4% and 81.2% for GF2 and GF4, respectively. This initial release from the AXSA films is less than that of previously reported pure AX films and higher than that of AX-GL films [32,33]. These results suggest that 10 cm (diameter) GF2 and GF3 films will release up to 21 mg GS in 24 h. Previous studies on GS-based wound dressing materials suggest that 0.1% GS is effective for wound infections [14]. Moreover, topical delivery of 0.1% GS is considered safe for avoiding the toxic effects (ototoxicity and nephrotoxicity) associated with systematic GS administration [41,51]. 

The drug release from the dressing material depends on various factors, such as the solubility of the drug in the release media, relaxation, swelling and erosion of the polymeric network, and diffusion rate of the drug [33,38,52]. Therefore, mathematical models were applied to investigate the GS release kinetics and mechanism of release from the GS-loaded AXSA films. The results are shown in Table 2. The higher regression coefficient “*R^2^*” values indicate the best fitting model. The results (Table 2) indicate that “*R^2^*” values, obtained by fitting zero-order (0.4498 and 0.4243), first-order (0.543 and 0.5856), and Higuchi (0.6804 and 0.7015) equations, were less than those obtained with the Korsmeyer–Peppas equation (0.9788 and 0.9451). These results suggest that the Korsmeyer–Peppas model is the best-fitted model (“*R*^2^” close to 1) for GS release from GF2 and GF3 films. The values of *n* (release exponent) for the Korsmeyer–Peppas model were 0.4603 and 0.4982 for GF2 and GF3 films, respectively. According to Korsmeyer–Peppas, for thin films, *n* values below 0.5 indicate that Fickian diffusion is the mechanism governing drug release [53]. Therefore, the *n* values of GS release from the AXSA films indicate that Fickian diffusion was the predominant mechanism, with the rate of GS release being limited by solvent penetration rate. A similar release mechanism was reported by Pires et al. for extract release from chitosan and alginate membranes intended for wound dressing [54]. These findings for the GS release profile suggest that the AXSA films hold potential for the treatment of infected wounds by delivering an initial high concentration of GS, followed by slower release up to 24 h. 

### 3.4. Antibacterial Effect of the Films

**Figure 6:**
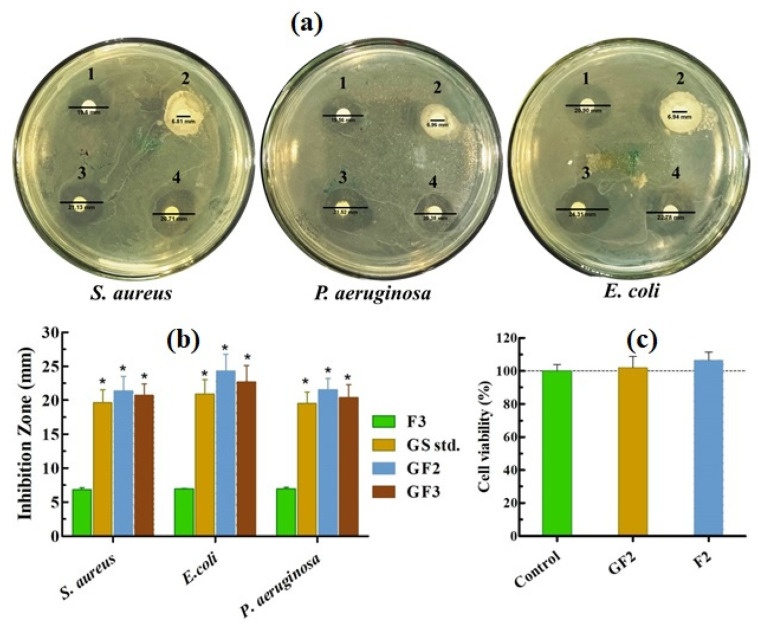
(**a**) Antibacterial effect of F3 (1), gentamicin sulphate (GS) (2), GF2 (3), GF3 (4); (**b**) inhibition zones and (**c**) cell viability of arabinoxylan (AX)-sodium alginate (SA) films (mean ± SD, *n* = 3). Asterisk (*) indicate statistically significant difference (*p* < 0.05) as compared to blank film (F3).

The results of the antibacterial effect of GS solution, F3, GF2, and GF3 films are depicted in Figure 6a,b. In this experiment, blank AXSA film (F3) was used as a negative control, and filter paper disks dipped in 0.1% (*w*/*w*) GS solution were used as a positive control. The F3 (blank) films formed no clear inhibition zones, suggesting an insignificant antibacterial effect of AXSA films against the tested bacterial strains. However, opaque regions were observed around F2 films due to bacterial growth in the expanded blank AXSA films. In contrast, positive control (GS std.) and GS-loaded AXSA films (GF2 and GF3) exhibited significant antibacterial effects against both Gram-positive (*S. aureus*) and Gram-negative (*E. coli and P. aeruginosa*) bacteria. The values of inhibition zones for GF2 and GF3 were slightly higher than for the positive control. The antibacterial effect of the films was slightly higher against *E. coli* compared to the other two test strains, but this difference was statistically insignificant. The antibacterial effect of the AXSA films was better than the effect of recently reported chitosan-SA/GS nanofibrous wound dressing [35]. These results indicate GS-loaded AXSA films’ potential to protect wounds against infections caused by Gram-positive and Gram-negative bacteria.

### 3.5. Indirect Cell Viability Assay

The indirect cell viability assay results of AXSA films against MRC-5 are depicted in Figure 6c. The results display that the viability of the cells treated with the extract of F2 and GF2 films exhibited no significant difference from that of untreated (control) cells. Cell viability assays are typically performed to predict films’ safety for application on living tissues [7]. The extracts of F2 and GF2 films demonstrated no cytotoxic effects on normal cells and did not decrease cell viability. The cell viability (%) of the tested AXSA films was higher than the previously reported AX films indicating that the biocompatibility of the films increased by combining AX with SA in film formulation [33]. Therefore, it can be suggested that AXSA films are safe for in vivo applications. 

## 4. Conclusions

The findings of this study demonstrate that the optimized AXSA films possess various desirable characteristics for antibacterial wound dressings. FTIR, XRD, and TGA analyses suggested that AX, SA, Gly, and GS were finely blended and formed hydrogen bonds. The prepared AXSA films were smooth, flexible, and transparent, which can aid in inspecting wounds without removing the dressing. The skin-like mechanical strength and flexibility of AXSA films make them suitable for their application on wounds in problematic body areas like joints. The moderate water uptake (expansion) and transmission (WVTR) of these films could be helpful for protecting moderately exudating wounds from dehydration and to maintain a moist environment for healing. The AXSA films released more than 80% of the loaded GS in 24 h with the initial rapid release (in the first hour), which can be used to control infections. The inhibition zones formed by the GS-loaded AXSA films in Gram-positive and Gram-negative bacteria were similar to those of GS solution, suggesting that these films can be used to control bacterial growth in infected wounds. The AXSA films also demonstrated excellent cytocompatibility in MTT assay, suggesting their safety for in vivo applications. Overall, we demonstrated that the AXSA films are a potential candidate for antibacterial wound dressing applications.

## Figures and Tables

**Table 1:** Formulation codes and composition of arabinoxylan-sodium alginate (AXSA) films.

Composition (% *w*/*w*)	F1	F2	F3	F4	GF1	GF2	GF3	GF4
Arabinoxylan (AX)	1.5	2	2.5	3	1.5	2	2.5	3
Sodium alginate (SA)	3.5	3	2.5	2	3.5	3	2.5	2
Glycerol (Gly)	2.5	2.5	2.5	2.5	2.5	2.5	2.5	2.5
Gentamicin (GS)	–	–	–	–	0.1	0.1	0.1	0.1
Distilled water	92.5	92.5	92.5	92.5	92.4	92.4	92.4	92.4

**Table 2:** Mathematical modeling of GS release from AXSA films.

Model	Parameters	GF2	GF3
Zero order	*R* ^2^	0.4498	0.4243
	*K*_0_	1.7521	1.6294
First order	*R* ^2^	0.543	0.5856
	*K*_1_	−0.0225	−0.0211
Higuchi	*R* ^2^	0.6804	0.7015
	*K_H_*	11.884	12.618
Korsmeyer–Peppas	*R* ^2^	0.9788	0.9451
	*K_K_*	1.6417	1.5627
	*n*	0.4603	0.4982

## Data Availability

Data sharing is not applicable to this article.

## Supplementary Materials

The following supporting information can be downloaded at: https://www.mdpi.com/article/10.3390/ijms23052899/s1.
