# Supplementary material for: Development and Characterization of Gentamicin-Loaded Arabinoxylan-Sodium Alginate Films as Antibacterial Wound Dressing
Source: Int J Mol Sci. 2022 Mar 7;23(5):2899. doi: 10.3390/ijms23052899 (PMC8911204; doi:10.3390/ijms23052899)
Supplement: Supplementary file 1 [file ijms-23-02899-s001.zip › ijms-1590672-supplementary.pdf]

Supplementary Material

Development and Characterization of Gentamicin Loaded Arabinoxylan-Sodium Alginate Films as Antibacterial Wound Dressing

Abdulaziz I. Alzarea[1], Nabil K. Alruwaili [2], Muhammad Masood Ahmad [2], Muhammad Usman Munir [3], Adeel Masood Butt [4], Ziyad A. Alrowaili [5], Muhammad Syafiq Bin Shahari [6], Ziyad S. Almalki [7], Saad S. Alqahtani [8], Anton V. Dolzhenko [6], and Naveed Ahmad [2 *]

[1] Department of Clinical Pharmacy, College of Pharmacy, Jouf University, Sakaka 72388, Aljouf, Saudi Arabia

[2] Department of Pharmaceutics, College of Pharmacy, Jouf University, Sakaka 72388, Aljouf, Saudi Arabia

[3] Department of Pharmaceutical Chemistry, College of Pharmacy, Jouf University, Sakaka 72388, Aljouf, Saudi Arabia

[4] Institute of Pharmaceutical Sciences, University of Veterinary & Animal Sciences, Lahore 54000, Pakistan

[5] Department of Physics, College of Sciences Jouf University, Sakaka 72388, Aljouf, Saudi Arabia

[6] School of Pharmacy, Monash University Malaysia, Jalan Lagoon Selatan, Bandar Sunway, Selangor Darul Ehsan 47500, Malaysia

[7] Department of Clinical Pharmacy, College of Pharmacy, Prince Sattam Bin Abdulaziz University, Al-Kharj 11942, Riyadh, Saudi Arabia

[8] Department of Clinical Pharmacy, College of Pharmacy, Jazan University, Jazan 45142, Saudi Arabia

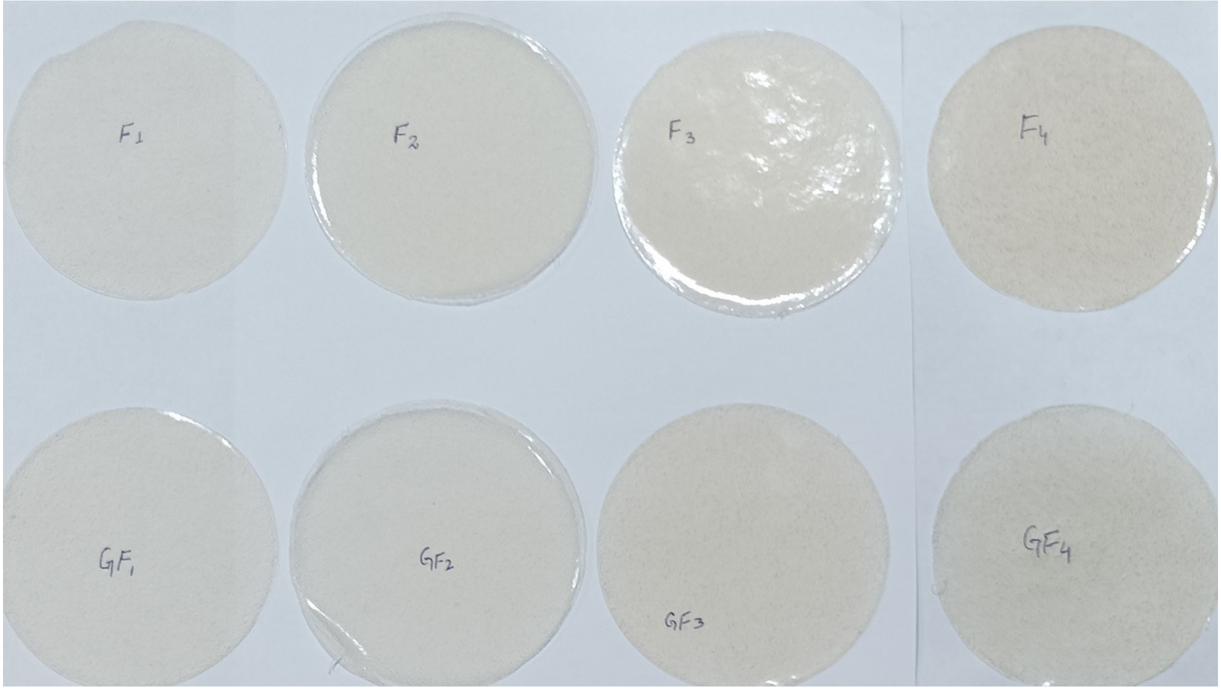

Figure S1. Optical images of blank (F1, F2, F3, and F4) and GS-loaded (GF1, GF2, GF2, and GF4) AXSA films.

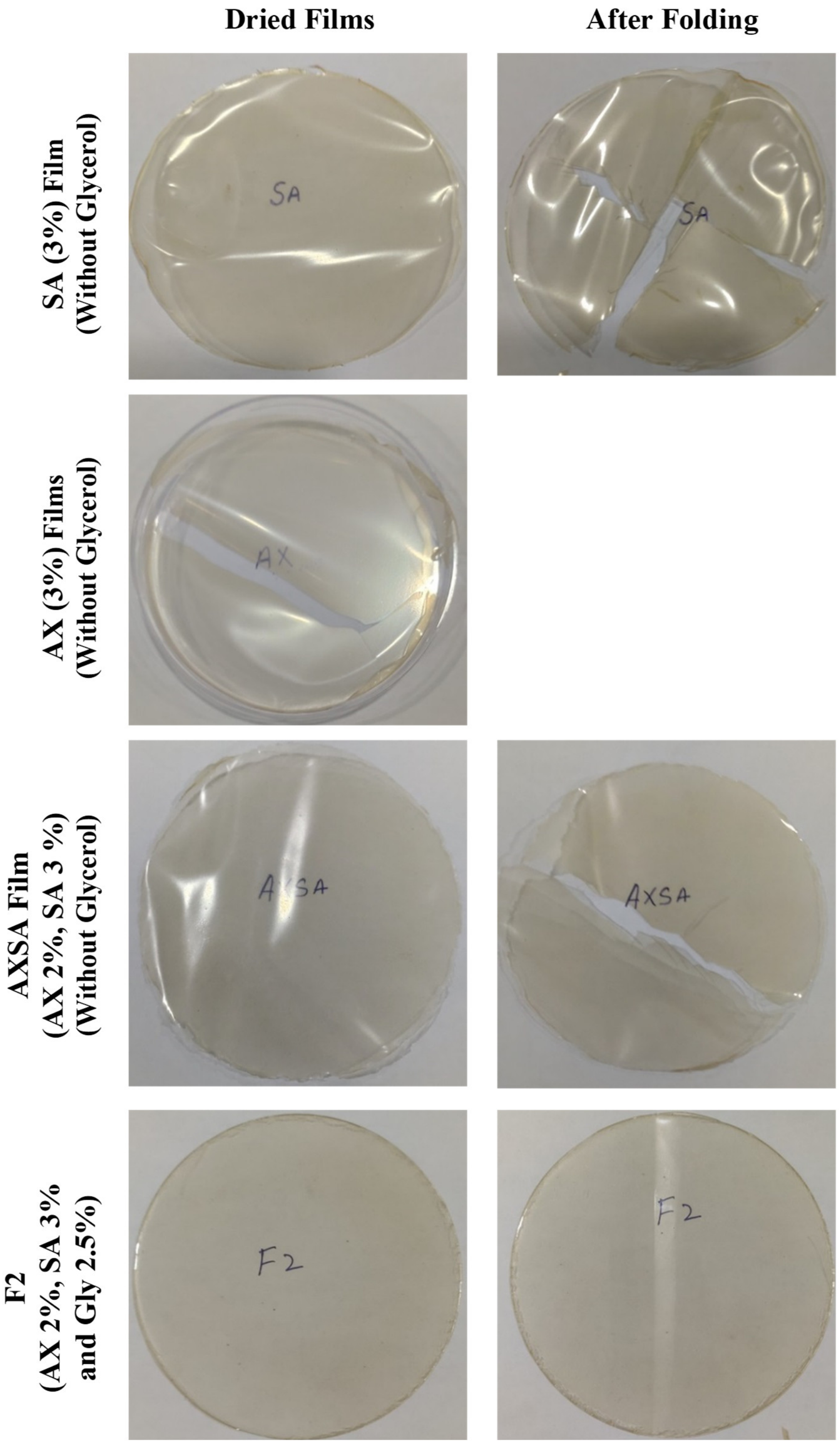

Figure S2. Comparison between the handling of AX, SA and AXSA films (without glycerol) with F2 films (with 2.5% glycerol).

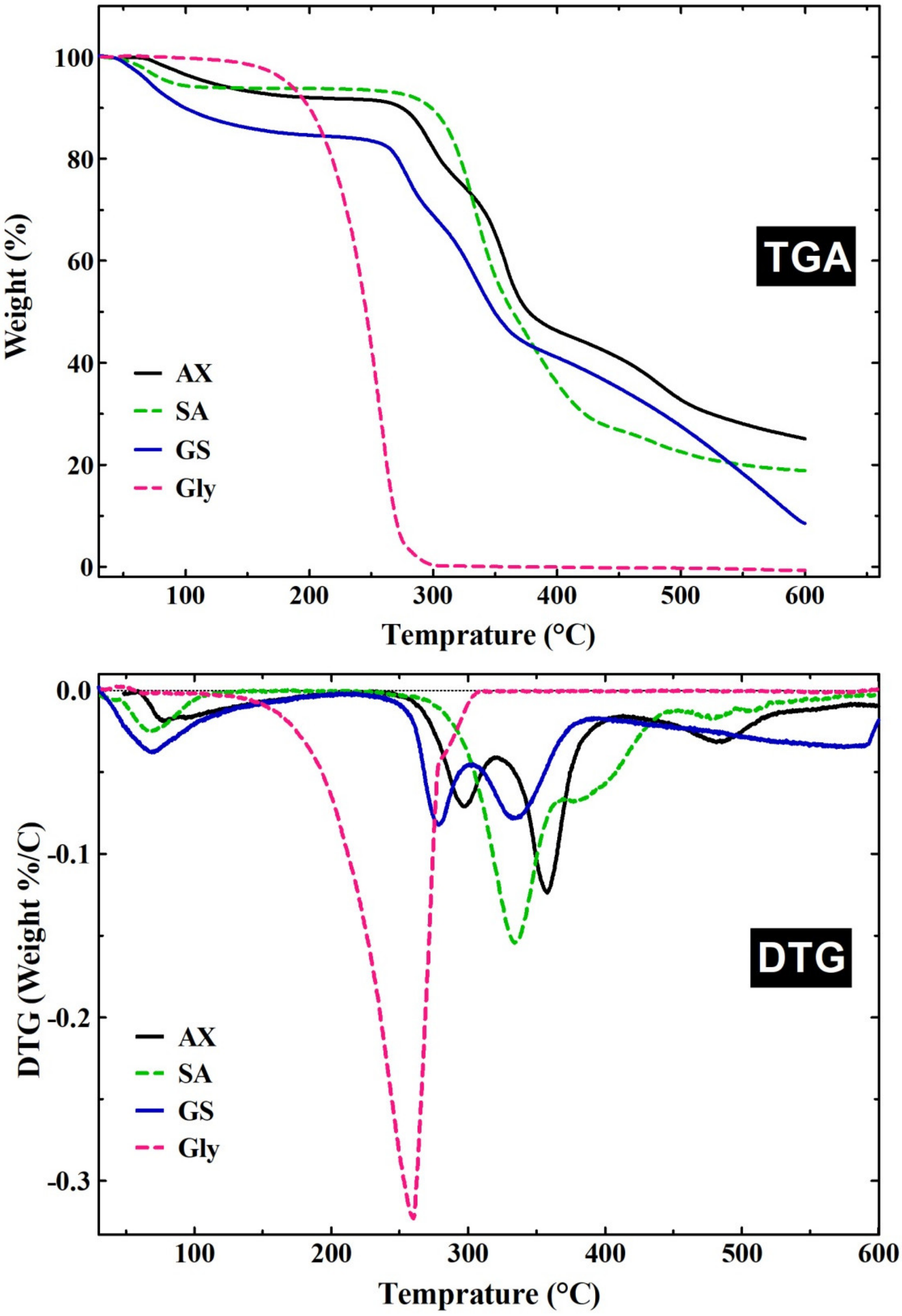

Figure S3. TG and DTG curves of of the AX, SA, GS and Gly.

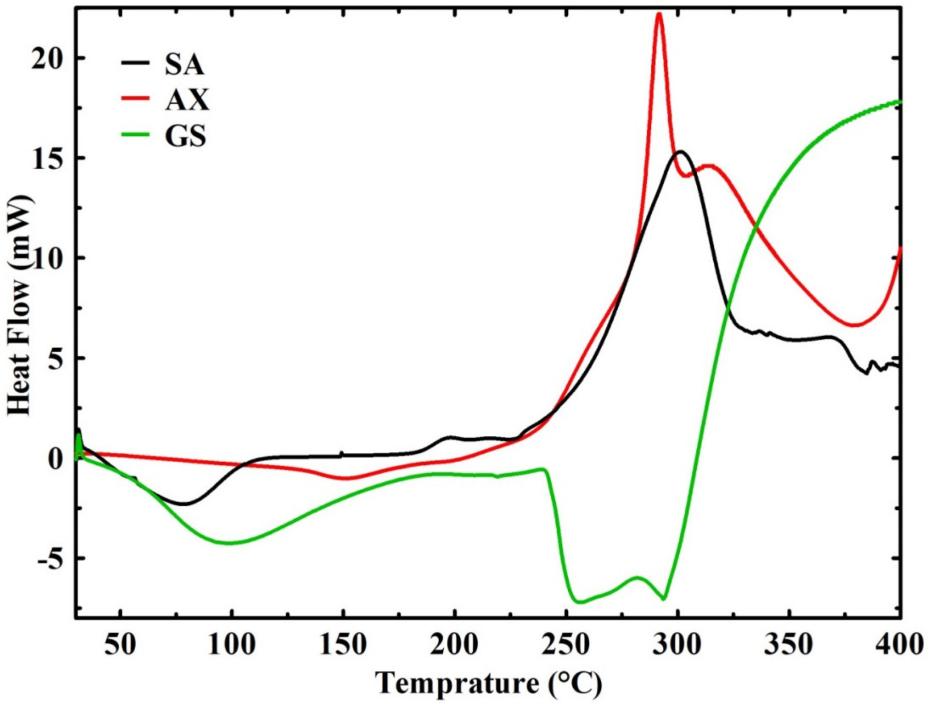

Figure S4. DSC curves of AX, SA, and GS powder.
